# Glucosamine protects against radiation‐induced lung injury via inhibition of epithelial‐mesenchymal transition

**DOI:** 10.1111/jcmm.15662

**Published:** 2020-07-22

**Authors:** Xiao Lei, Na Ma, Yanjie Liang, Junyan Liu, Pei Zhang, Yanan Han, Wei Chen, Lehui Du, Baolin Qu

**Affiliations:** ^1^ Department of Radiation Oncology The First Medical Center of Chinese PLA General Hospital Beijing China; ^2^ Department of General Surgery The First Medical Center of Chinese PLA General Hospital Beijing China; ^3^ Facilities and Support Center Academy of Military Medical Sciences Beijing China

**Keywords:** EMT, glucosamine, lung tissues, radiation

## Abstract

Radiotherapy is one of the most important treatments for chest tumours. Although there are plenty of strategies to prevent damage to normal lung tissues, it cannot be avoided with the emergence of radiation‐induced lung injury. The purpose of this study was to investigate the potential radioprotective effects of glucosamine, which exerted anti‐inflammatory activity in joint inflammation. In this study, we found glucosamine relieved inflammatory response and structural damages in lung tissues after radiation via HE staining. Then, we detected the level of epithelial‐mesenchymal transition marker in vitro and in vivo, which we could clearly observe that glucosamine treatment inhibited epithelial‐mesenchymal transition. Besides, we found glucosamine could inhibit apoptosis and promote proliferation of normal lung epithelial cells in vitro caused by radiation. In conclusion, our data showed that glucosamine alleviated radiation‐induced lung injury via inhibiting epithelial‐mesenchymal transition, which indicated glucosamine could be a novel potential radioprotector for radiation‐induced lung injury.

## 1 INTRODUCTION

Radiation‐induced lung injury often occurs with radiotherapy for chest tumours. Nowadays, radiotherapy is one of the most common treatment methods for chest tumours, however, side effects caused by radiotherapy raised more and more attention.^1^ Radiation‐induced lung injury can be divided into radiation pneumonitis and radiation fibrosis according to its development process. The main clinical manifestations of radiation‐induced lung injury are inflammatory infiltration of the alveolar interstitial substance, progressive dyspnea, worsening lung function, and eventually leading to respiratory failure.^2^ The occurrence of radiation‐induced lung injury has greatly reduced the prognosis survival rate of patient.^3^ Therefore, the protection of radiation‐induced lung injury caused by radiotherapy has extremely important medical significance.

The cause of radiation‐induced lung injury has not been affirmed in academia. Most research shows that the reason might be the imbalance of various inflammatory cells, fibroblasts and related cytokines induced by radiation, resulting in excessive proliferation and migration of fibroblasts and extracellular interstitial (ECM) deposition, which has caused damage to the body.^4^ At present, there are little effective protective and therapeutic drugs for radiation‐induced lung injury. Traditional radioprotective drugs provide a certain protective effect on normal lung tissues while exerting radioprotective effects on tumour cells. The characteristics of this type of drugs greatly reduce the effect of tumour radiotherapy, which restricts its application in chest radiotherapy for clinical tumour patients.^5^ Therefore, it is urgent to find a new protective drug with significant curative effect, low toxic and side effects in the clinical treatment of radiation‐induced lung injury.

Glucosamine is a compound in which one hydroxyl group of glucose is substituted with one amino group. It is a substance synthesized in the human body, which is an important nutrient for forming chondrocytes, and a natural tissue component of healthy joint cartilage.^6^ Extensive medical research in the United States, Europe and Japan has shown that glucosamine can help repair and maintain cartilage, and stimulate the growth of chondrocytes.^7^ Studied shows that glucosamine could reduce prostaglandin E2 (PGE2) production and interfere with nuclear factor kappa B.^8^ Furthermore, it shows great anti‐inflammatory and free radical scavenging effects.^9^ According to its features, we have been suggested whether it could alleviate radiation‐induced lung injury. In this study, we found glucosamine effectively protected against radiation‐induced lung injury through epithelial‐mesenchymal transition, which provided a novel potential radioprotector for radiation‐induced lung injury.

## 2 MATERIALS AND METHODS

### 2.1 Cell culture and glucosamine treatments

Rat lung epithelial‐T‐antigen negative (RLE‐6TN) cell was obtained from ATCC (USA). This cell line was maintained in RPMI‐1640 with 10% foetal bovine serum at 37°C in a 5% CO2 humidified chamber. RLE‐6TN cells were treated with different concentration of glucosamine 3 hours before radiation.

### 2.2 Mice and glucosamine treatments

Eight‐week‐old female wild‐type C57BL/6 mice were obtained from the Experimental Animal Center of Chinese Academy of Sciences, Shanghai, China, were used for the animal experiment. All the methods were approved by the Animal Ethics Committee of Second Military Medical University, with the Guide for Care and Use of Laboratory Animals published by the US National Institute of Health (publication no. 96‐01). Mice were fed in daily changed individual cages, at 25 ± 1°C with sufficient sterilized mice food and water. Glucosamine was administered by intraperitoneal injection 3 days before irradiation at the dose of 150 mg/kg/day. WR2721(100 mg/kg/day) and saline were given through intraperitoneal injection at the same time. At different time points after radiation exposure, mice were sacrificed for the following experiments to explore the radioprotective effects of glucosamine.

### 2.3 Irradiation

Sixty Co γ‐rays was used in this study (Faculty of Naval Medicine, Second Military Medical University, Shanghai, China). After anesthetization with 10% chloral hydrate (350 mg/kg), the mice were treated with local irradiation in lung. The mice received total‐lung irradiation in a holder designed to immobilize unanaesthetized mice so that the lung was exposed to the beam.^10^ The radiation dose was 15 Gy and dose rate was 1Gy/min. Cells were exposed with different dose depending on different demands.

### 2.4 Tissue staining

HE and Immunohistochemistry staining were used to explore the lung tissue changes. At certain time points after local radiation, the mice were sacrificed and lung tissues were isolated, fixed. Anti‐Vimentin (1:500, Cell Signaling Tech.), anti‐E‐cadherin (1:500, Cell Signaling Tech.) and anti‐a‐SMA (1:500, Cell Signaling Tech.) were utilized to stain in tissue sections. We used the fluorescence microscope (Nikon Eclipse Ti‐SR, Nikon) to obtain the images.

### 2.5 Western blot analysis

Different group samples were prepared to extract the protein (lung tissues and cells). E‐cadherin (1:1000; Cell Signaling Tech.), Vimentin (1:1000; Cell Signaling Tech.), a‐SMA (1:1000; Cell Signaling Tech.), actin (1:1000; Cell Signaling Tech.) were used to explore the changes in epithelial‐mesenchymal transition. The secondary antibody (1:5000) was purchased from Abcam.

### 2.6 Cell viability and apoptosis assay

CCK‐8 assay and clonogenic assay were performed to assess optimal concentration of glucosamine. 24 hours and 48 hours after drug treatment, cells were dealt with an CCK‐8 kit (Invitrogen), 1 hours later cells were counted by absorbance measurements at 450 nm. For clonogenic assay, cells were seeded into a 6‐well plate and irradiated with 0, 2, 4, 8 Gy. After almost 2 weeks' incubation, cells were fixed with paraformaldehyde and stained with 1% methylene blue. After 30 minutes, the cells were washed with PBS and colony formation could be counted. For apoptosis assay, cells' apoptosis was analysed using the apoptosis detection kit (Invitrogen) by dual staining of Annexin V‐FITC and Propidium Iodide (PI). Then cell apoptosis was analysed by flow cytometry (Beckman Cytoflex).

### 2.7 Statistical analysis

Data were expressed as mean ± standard error of the mean. One‐way ANOVA was used to test group differences. The differences between the two groups were analysed via two‐tailed Student's *t* test. *P* < .05 was considered statistically significant.

## 3 RESULTS AND DISCUSSION

**FIGURE 1:**
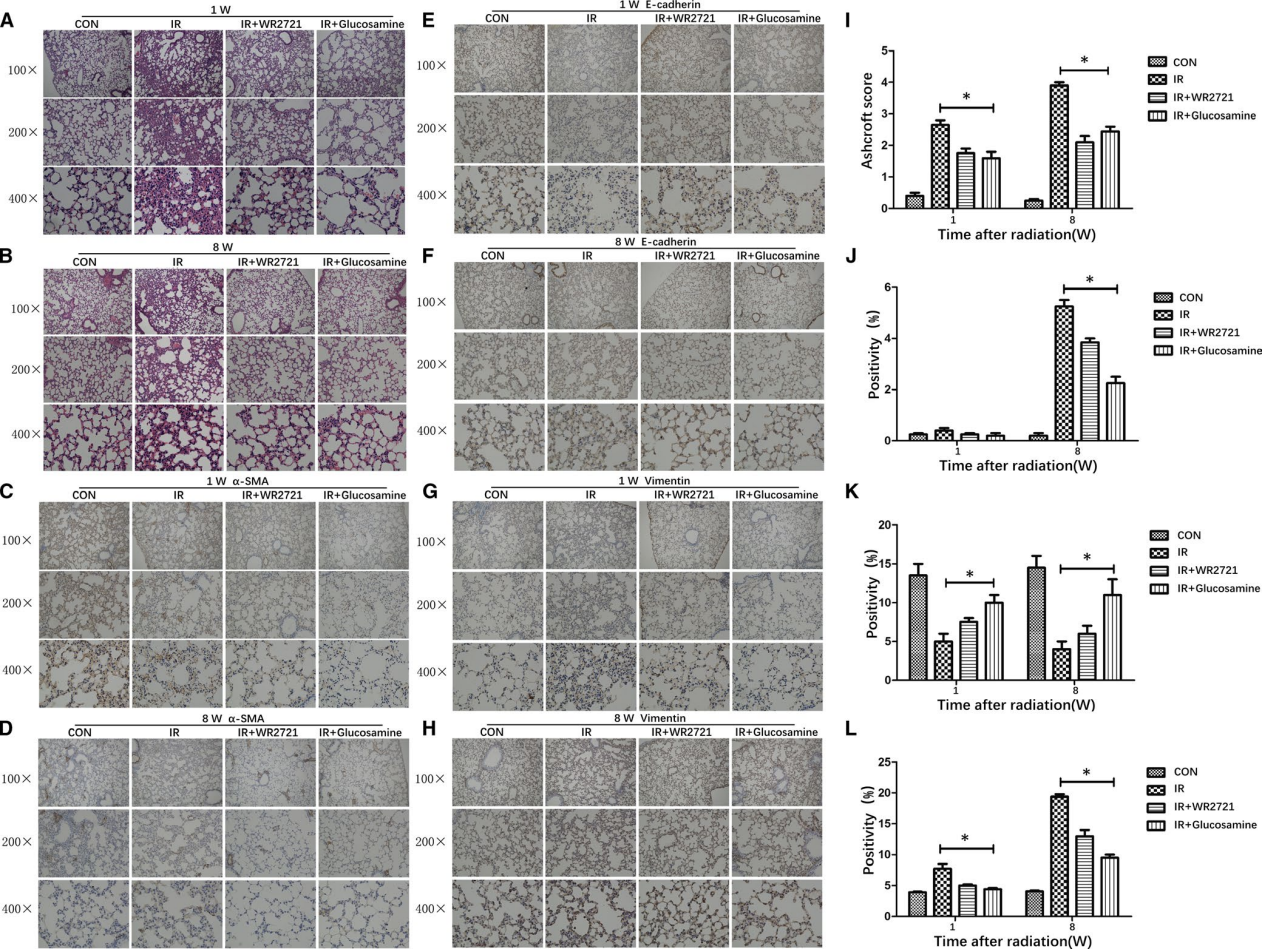
Glucosamine relieved inflammatory response and structural damages and impeded radiation‐induced epithelial‐mesenchymal transition in lung tissues after IR. A, Representative images of HE staining of lung tissue sections at 1 wk post‐irradiation. B, Representative images of HE staining of lung tissue sections at 8 wk post‐irradiation. C, Representative images of α‐SMA staining of lung tissue sections at 1 wk post‐irradiation. D, Representative images of α‐SMA staining of lung tissue sections at 8 wk post‐irradiation. E, Representative images of E‐cadherin staining of lung tissue sections at 1 wk post‐irradiation. F, Representative images E‐cadherin staining of lung tissue sections at 8 wk post‐irradiation. G, Representative images of Vimentin staining of lung tissue sections at 1 wk post‐irradiation. H, Representative images Vimentin staining of lung tissue sections at 8 wk post‐irradiation. I, A bar graph of Ashcroft scoring of HE staining of lung tissues. J, A bar graph showing quantification analysis of α‐SMA positive cells in slide from lung tissues. K, A bar graph showing quantification analysis of E‐cadherin positive cells in slide from lung tissues. L, A bar graph showing quantification analysis of Vimentin positive cells in slide from lung tissues. *P < .05 vs single radiation group

It has been proved that glucosamine exerted anti‐inflammatory activity in joint inflammation.^11^ In order to determine whether glucosamine conferred to anti‐inflammatory activity in lung tissues after radiation, we used mouse lung local radiation model. After 15Gy local irradiation, we collected lung tissues at 1 week and 8  week. Then, we did the sections of lung tissues with HE staining. As we could see in Figure 1A,B and I, alveolar wall structure was normal in the control group. 1 week after the irradiation, the IR group showed thickening of the alveolar septum and a large amount of inflammatory cell infiltration. By 8 weeks after irradiation, the alveolar wall thickened and aggravated, the alveoli collapsed to varying degrees, the fibroblasts increased significantly, and the alveolar wall showed fibrosis to varying degrees. By comparing the IR group with the glucosamine‐treated group, it could be clearly found that glucosamine exhibited inflammatory inhibitory effect in lung tissue exposure, which meant glucosamine might have the great anti‐radiation effect in lung tissues. In the WR2721 treatment group, the mouse lung tissue also showed a milder inflammatory response.

**FIGURE 2:**
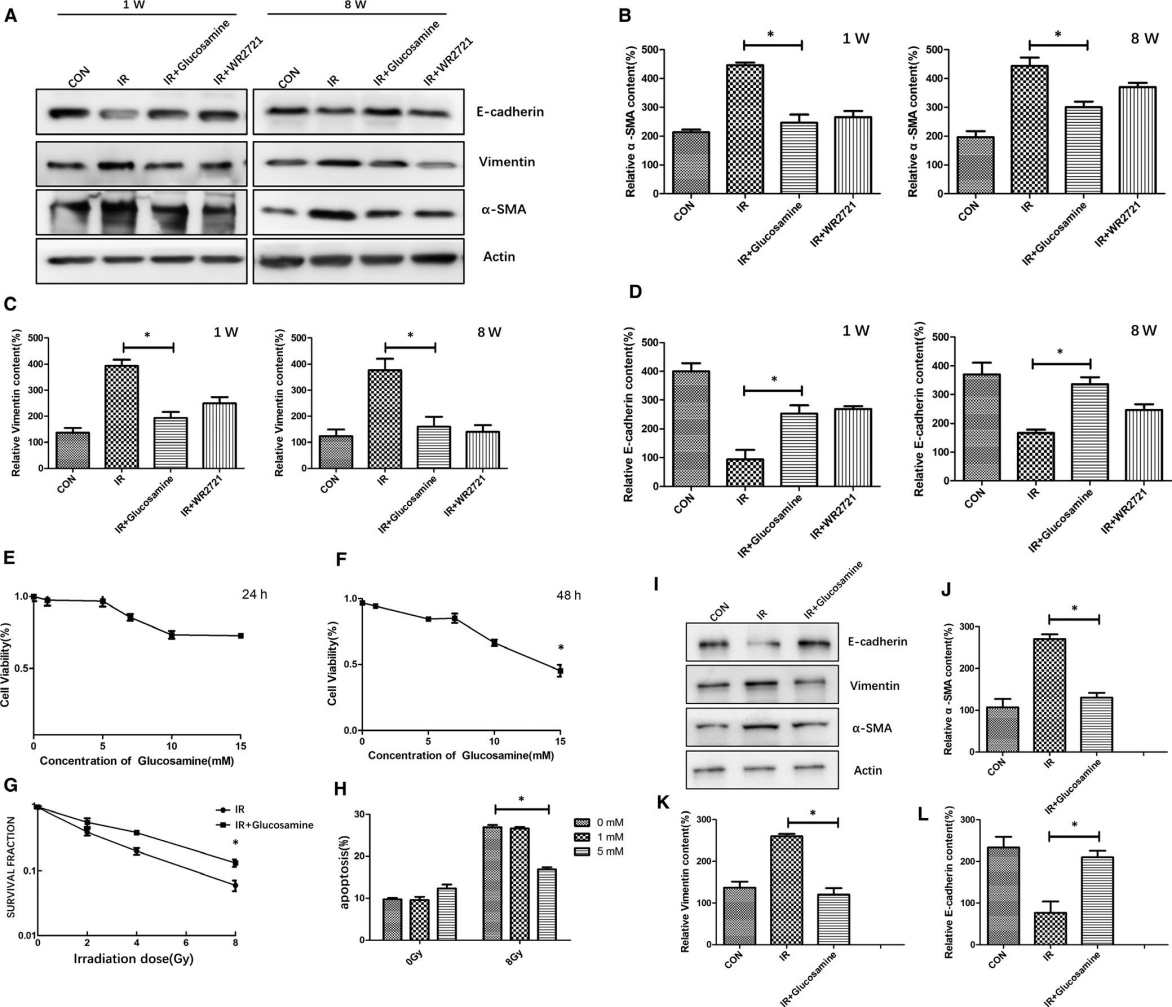
Glucosamine protected normal lung epithelial cells from IR and inhibited radiation‐induced epithelial‐mesenchymal transition in vitro. A, The contents of EMT markers were tested in lung tissues from different groups. (B‐D). Qualification of protein expression levels of α‐SMA, Vimentin and E‐cadherin in different groups. *P < .05 E, Cell viability was tested with different concentration of glucosamine at 24 h after radiation. F, Cell viability was tested with different concentration of glucosamine at 48 h after radiation. G, RLE‐6TN treated with or without glucosamine(5 mmol/L) was analysed for their colony‐forming ability against radiation. H, RLE‐6TN treated with or without glucosamine (1 mmol/L, 5 mmol/L) was analysed for apoptosis by Flow cytometric analysis against radiation. *P < .05 I, The contents of EMT markers were tested in RLE‐6TN treated with or without glucosamine(5 mmol/L). (K‐L). Qualification of protein expression levels of α‐SMA, Vimentin and E‐cadherin in different groups. *P < .05

Recent studies showed that radiation could induce epithelial‐mesenchymal transition (EMT), which was closely linked to the development of pulmonary fibrosis.^12^ To fully confirm the role of glucosamine in lung tissues after radiation, we utilized immunofluorescence staining to check the changes of epithelial marker (E‐cadherin) and interstitial markers (α‐SMA and Vimentin) in lung tissues at 1 week and 8  week after radiation. Figure 1C‐H,J‐L showed that glucosamine could significantly inhibit the overexpression of interstitial markers α‐SMA and Vimentin, and inhibit the down‐regulation of interstitial markers E‐cadherin caused by radiation. The effect of WR2721 was weaker than glucosamine treatment. We also tested the content of related markers in lung tissues at 1 week and 8 weeks after radiation (Figure 2A‐D), which showed the consistent results with immunofluorescence staining.

To further explore the role of glucosamine in radiation‐induced lung injury, we did some in vivo experiments. In order to choose the optimal concentration of the glucosamine in normal lung epithelial cells, firstly we did the CCK‐8 assay at 24 hours and 48 hours after glucosamine treatment, we found glucosamine at the concentration less than 10 mmol/L did not inhibit the growth and proliferation of RLE‐6TN cells (Figure 2E, F). Then we chose glucosamine at the concentration 5 mmol/L for the clonogenic assay, our data showed that glucosamine could significantly reduce the cell death of RLE‐6TN cells induced by radiation (Figure 2G). Furthermore, the apoptosis assay showed that glucosamine at the concentration 5 mmol/L significantly reduced apoptosis of RLE‐6TN cells after IR (Figure 2H). To fully explain the effect of glucosamine in lung tissues after radiation, we further tested the content of epithelial‐mesenchymal transition (EMT) markers which induced via radiation in vitro. The results showed that glucosamine at the concentration 5 mmol/L could significantly inhibit the overexpression of α‐SMA and Vimentin, and inhibit the down‐regulation of E‐cadherin induced by radiation in RLE‐6TN cells(Figure 2I‐L).

At present, glucocorticoid is mainly used in clinical to relieve the acute inflammatory response of radiation‐induced lung injury, but it has no relief effect on long‐term radiation‐induced pulmonary fibrosis.^13^ At the same time, its serious side effects have limited the scope and dosage of the drug for clinical application. In animal experiment, the radioprotective drug WR‐2721 can effectively prevent and reduce the acute inflammatory response to radiation‐induced lung injury. However, it has no obvious effect on long‐term radioactive pulmonary fibrosis and has relatively strong biological toxicity, which limits its clinical application.^14^ Therefore, it is essential to find a novel safe and effective radioprotective drug for radiation‐induced lung injury.

In our study, we found glucosamine effectively alleviated inflammatory response and structural damages in lung tissues after radiation. It could also inhibit apoptosis and promote proliferation of normal lung epithelial cells in vitro caused by radiation. Considering it low toxicity, we think it is a novel safe and effective radioprotective drug for radiation‐induced lung injury. Further we explored the changes of epithelial‐mesenchymal transition markers after radiation in vivo and in vitro, our data showed that glucosamine could inhibit epithelial‐mesenchymal transition caused by radiation, which contributed to its radioprotective effects.

Epithelial‐mesenchymal transition (EMT) is one of the important causes of pulmonary fibrosis. It plays critical roles in the progression of radiation‐induced lung injury. It is characterized by down‐regulation of E‐cadherin and up‐regulation of Vimentin and α‐SMA.^15^ Our data showed that glucosamine treatment could significantly inhibit the overexpression of interstitial markers α‐SMA and Vimentin, and reduce the down‐regulation of interstitial markers E‐cadherin caused by radiation in vivo and in vitro, which clearly indicated glucosamine could inhibit epithelial‐mesenchymal transition process in radiation‐induced lung injury, which alleviated the symptoms of radiation‐induced lung injury.

In summary, our data showed that glucosamine could alleviate radiation‐induced lung injury. The underlying mechanism might be associated with the inhibition of epithelial‐mesenchymal transition process, which indicated glucosamine could be a novel potential radioprotector for radiation‐induced lung injury.

## CONFLICTS OF INTEREST

The authors have no conflicts of interest to disclose.

## AUTHOR CONTRIBUTION

**Xiao Lei:** Conceptualization (lead); Data curation (lead); Writing‐original draft (lead); Writing‐review & editing (lead). **Na Ma:** Investigation (equal); Supervision (equal). **Yanjie Liang:** Project administration (equal); Validation (equal). **Junyan Liu:** Formal analysis (equal); Resources (equal). **Pei Zhang:** Conceptualization (equal); Resources (equal); Software (equal). **Yanan Han:** Methodology (equal); Visualization (equal). **Wei Chen:** Data curation (equal); Writing‐original draft (equal). **Lehui Du:** Formal analysis (equal); Investigation (equal); Writing‐original draft (equal); Writing‐review & editing (equal). **Baolin Qu:** Project administration (lead); Writing‐original draft (lead); Writing‐review & editing (equal).

## Data Availability

The data sets are available under reasonable request.
